# Supplementary material for: Recent centennial drought on the Tibetan Plateau is outstanding within the past 3500 years
Source: Nat Commun. 2025 Feb 3;16:1311. doi: 10.1038/s41467-025-56687-z (PMC11790959; doi:10.1038/s41467-025-56687-z)
Supplement: Supplementary file 1 — Supplementary Information [file 41467_2025_56687_MOESM1_ESM.pdf]

Supplementary information

Recent centennial drought on the Tibetan Plateau is outstanding within the past 3500 years

Authors: Yu Liu[1,2]*, Huiming Song[3]*, Zhisheng An[1,2], Qiang Li[3], Steven W. Leavitt[4], Ulf Büntgen[5,6,7], Qiufang Cai[1,2], Ruoshi Liu[3], Congxi Fang[8], Changfeng Sun[3], Kerstin Treydte[9,10], Meng Ren[11], Lidong Mo[12], Yi Song[1], Wenju Cai[13], Quan Zhang[1], Weijian Zhou[1,2], Achim Bräuning[14], Jussi Grießinger[14,15], Deliang Chen[16], Hans W. Linderholm[16], Ashish Sinha[17], Hai Cheng[3], Lu Wang[18], Ying Lei[1], Junyan Sun[1], Wei Gong[19], Xuxiang Li[3], Linlin Cui[20], Liang Ning[21,22,23], Lingfeng Wan[24], Thomas W. Crowther[12], Constantin M. Zohner[12]

Affiliations:

[1] The State Key Laboratory of Loess and Quaternary Geology, Institute of Earth Environment, Chinese Academy of Sciences, Xi'an, China.

[2] CAS Center for Excellence in Quaternary Science and Global Change, Chinese Academy of Sciences, Xi'an, China.

[3] Institute of Global Environmental Change, Xi'an Jiaotong University, Xi'an, China.

[4] The Laboratory of Tree-Ring Research, The University of Arizona, Tucson, AZ, USA.

[5] Department of Geography, University of Cambridge, Cambridge CB2 3EN, UK.

[6] Global Change Research Institute (CzechGlobe), Czech Academy of Sciences, 603 00 Brno, Czech Republic.

[7] Department of Geography, Faculty of Science, Masaryk University, 611 37 Brno, Czech Republic.

[8] Institute of Mountain Hazards and Environment, Chinese Academy of Sciences, Chengdu, China.

[9] Research Unit Forest Dynamics, Swiss Federal Research Institute (WSL), 8903 Birmensdorf, Switzerland.

[10] Oeschger Centre for Climate Change Research, University of Bern, 3012 Bern, Switzerland.

[11] Xi'an Institute for Innovative Earth Environment Research, Xi'an China.

[12] Institute of Integrative Biology, ETH Zurich (Swiss Federal Institute of Technology), Zurich, Switzerland.

[13] Centre for Southern Hemisphere Ocean Research (CSHOR), CSIRO Oceans and Atmosphere, Hobart, Australia.

[14] Institute of Geography, Friedrich-Alexander-University Erlangen-Nürnberg, 91058 Erlangen, Germany.

[15] University of Salzburg, Department of Environment and Biodiversity, 5020 Salzburg, Austria.

[16] Department of Earth Sciences, University of Gothenburg, Gothenburg, Sweden.

[17] Department of Earth Science, California State University, Dominguez Hills, Carson, CA, USA.

[18] Institute of Subtropical Agriculture, Chinese Academy of Sciences, Changsha, China.

[19] School of Archaeology and Museology, Peking University, Beijing, China.

[20] College of Atmospheric Sciences, Chengdu University of Information Technology, Chengdu, China.

[21] Key Laboratory for Virtual Geographic Environment, Ministry of Education, School of Geography, Nanjing Normal University, Nanjing, China.

[22] State Key Laboratory Cultivation Base of Geographical Environment Evolution of Jiangsu Province, School of Geography, Nanjing Normal University, Nanjing, China.

[23] Jiangsu Center for Collaborative Innovation in Geographical Information Resource Development and Application, School of Geography, Nanjing Normal University, Nanjing, China.

[24] Institute for Advanced Ocean Study (IAOS), Ocean University of China, Qingdao, China.

*Corresponding authors. Email: liuyu@loess.llqg.ac.cn (Yu Liu);
songhm@xjtu.edu.cn (Huiming Song)

Supplementary Note: Missing offsets among trees in our study

In designing our research plan, we carefully considered the initial sample selection. Our dataset comprises 17 samples, including 12 from living and dead trees at the SG site, spanning from 761 BC to AD 2010, and one sample from the DL site, covering AD 1082–1974. To extend the chronology, we also included four samples from ancient tombs, a common approach in tree-ring research when such wood is available [1, 2, 3]. Therefore, the SG samples constitute the primary dataset for this chronology. If the DL and archaeological samples are excluded, the remaining chronology from the SG site samples alone is highly correlated with the complete dataset (r=0.975, 761 BC–2010 AD) (Supplementary Fig. 9). Despite a 200 km distance between sample locations, no significant discrepancies were observed. Therefore, we retained the DL sequence in our overall chronology. Including additional samples would further strengthen the robustness of our findings. It is important to emphasize that long-term climate changes can cause variations in absolute $\delta^{18}O$ values in trees, making it inappropriate and in our sense meaningless to compare absolute tree-ring $\delta^{18}O$ values across different time spans. Therefore, our analysis of the altitude-isotope relationship was limited to only seven trees within the most recent common period from AD 1835–1974, further demonstrating the absence of altitude-related effects on tree-ring $\delta^{18}O$ values in our study region (Supplementary Fig. 10).

Although the archaeological wood used in our study was excavated from ancient tombs located at lower elevations, the juniper forests from which the trees originate grew on slopes with elevations between 3500–4100 m a.s.l. [4]. As a result, this wood likely shares similar elevation characteristics with the living and dead trees from SG. Additionally, elevation differences among SG samples are minimal (around 300 meters), indicating that any elevation effect on $\delta^{18}O$ values is negligible.

The small range in tree-ring $\delta^{18}O$ values across different sites can be attributed to the uniform characteristics of $\delta^{18}O$ and the relatively uniform terrain surrounding the Qaidam Basin. Unlike tree-ring width, which can vary locally, $\delta^{18}O$ values typically reflect broader atmospheric circulation patterns [1, 3, 5]. Despite the overall complexity of the Tibetan Plateau, the terrain on the northeastern edge of the Qaidam Basin is relatively uniform. Given this, 200 km is a relatively minor distance in the context of our study region's size. Therefore, similar elevation and terrain characteristics contribute to the consistent $\delta^{18}O$ values observed across samples from different sources. Consequently, latitude and distance do not significantly affect the $\delta^{18}O$ values of trees from various locations in this study.

Supplementary figures

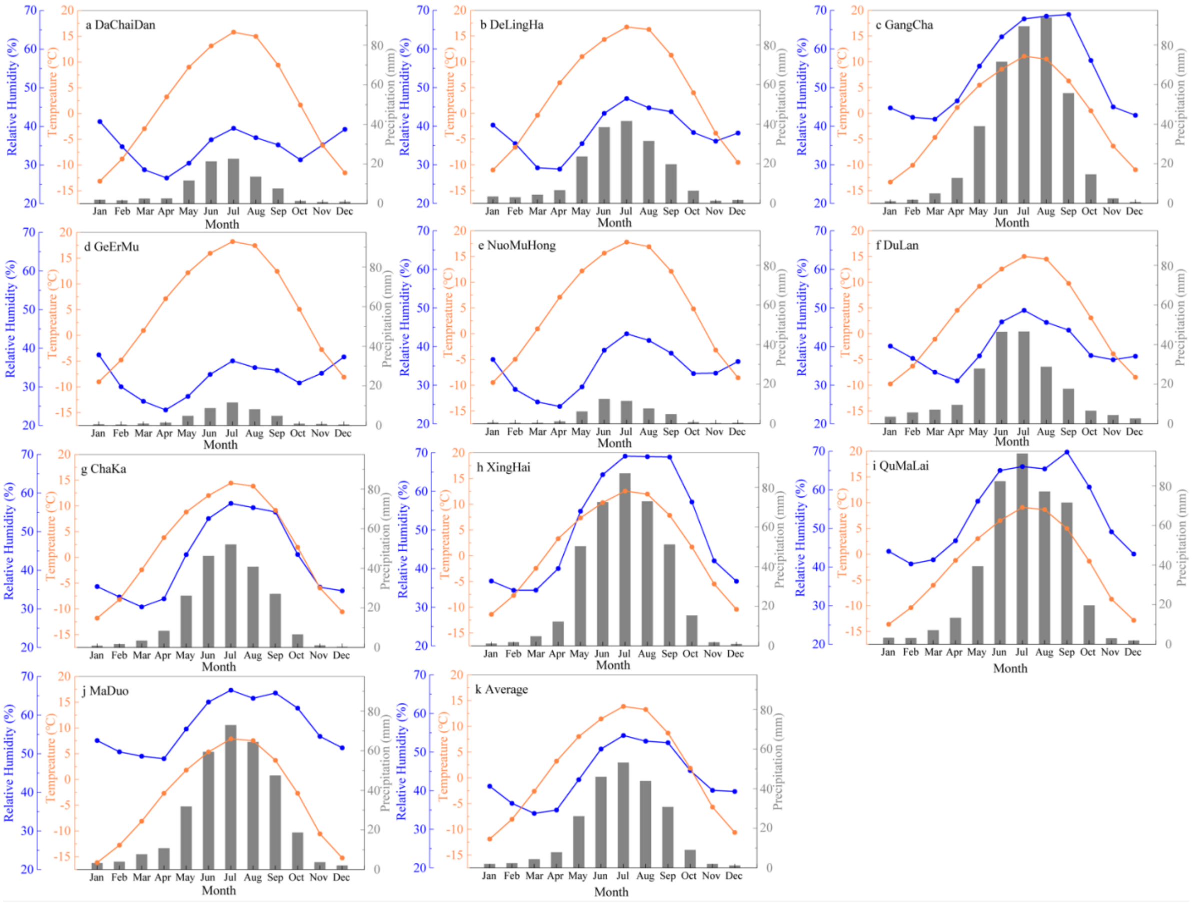

Supplementary Fig. 1. **Observed monthly total precipitation (grey bar), mean temperature (orange lines), and mean relative humidity (blue lines) for ten meteorological stations close to the study site (a–j) and for a scaled regional climate data (k) during AD 1960–2020.** The ten climate stations are as followed: DaChaiDan (37°51′N, 95°22′E, 3173.2 m a.s.l., AD 1956–2020), DeLingHa (37°22′N, 97°22′E, 2981.5 m a.s.l., AD 1955–2020), GangCha (37°20′N, 100°08′E, 3301.5 m a.s.l., AD 1957–2020), GeErMu (36°25′N, 94°54′E, 2807.6 m a.s.l., AD 1955−2020), NuoMuHong (36°26′N, 96°25′E, 2790.4 m a.s.l., AD 1956–2020), DuLan (36°18′N, 98°06′E, 3191.1 m a.s.l., AD 1954–2020), ChaKa (36°47′N, 99°05′E, 3087.6 m a.s.l., AD 1955–2020), XingHai (35°35′N, 99°59′E, 3323.2 m a.s.l., AD 1960–2020), QuMaLai (34°08′N, 95°47′E, 4175.0 m a.s.l., AD 1956–2020) and MaDuo (34°55′N, 98°13′E, 4272.3 m a.s.l., AD 1953–2020).

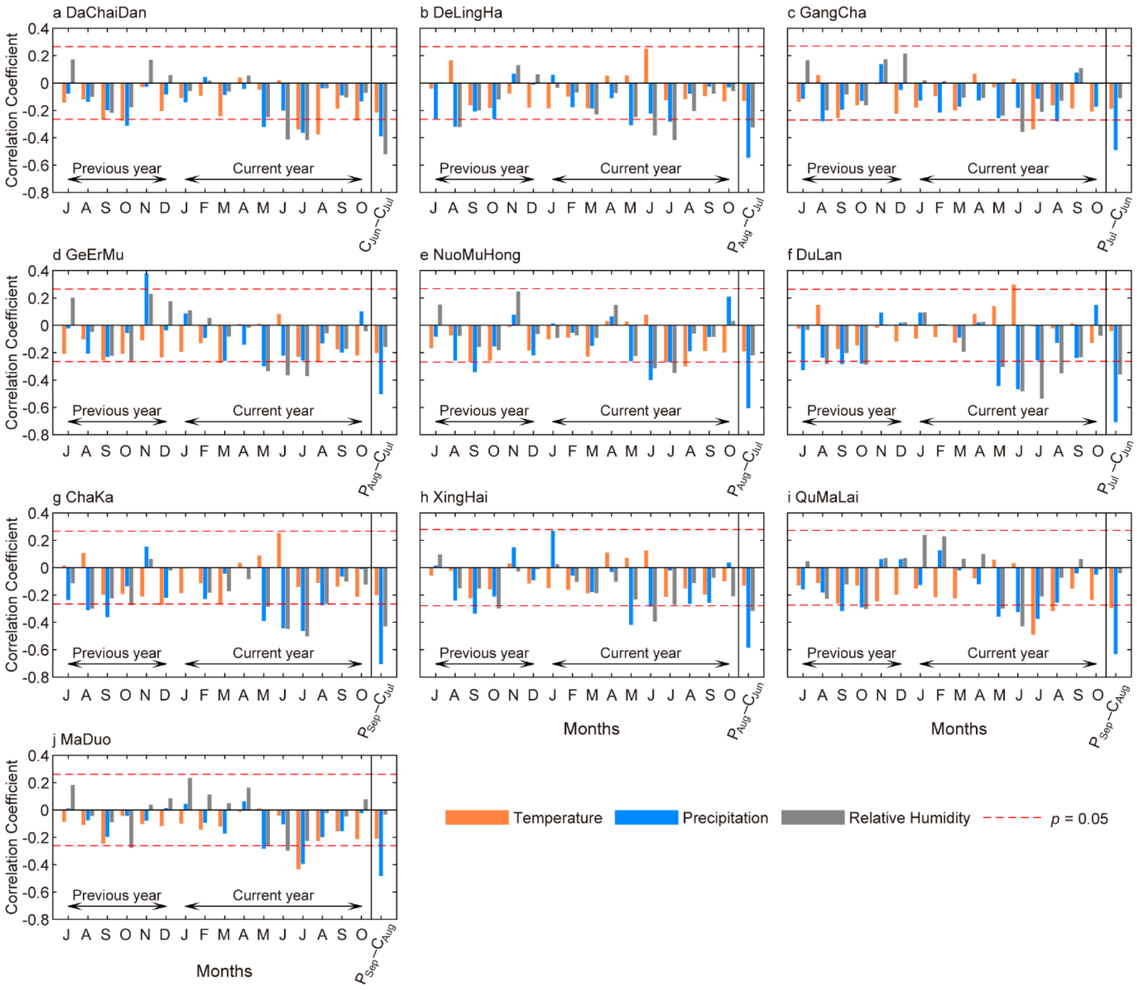

Supplementary Fig. 2. **Pearson's correlation coefficients calculated between the Tibetan Plateau tree-ring $\delta^{18}O$ chronology and different climate parameters for each of the ten nearby meteorological stations (a–j) for the period of previous July to current October.** The bars to the far right of each panel denote correlations with the total precipitation of the hydrological year (previous September to current August), showing the highest correlation with the Tibetan Plateau tree-ring $\delta^{18}O$ chronology. "P" denotes months in the previous year, "C" denotes months in the current year of tree-ring formation.

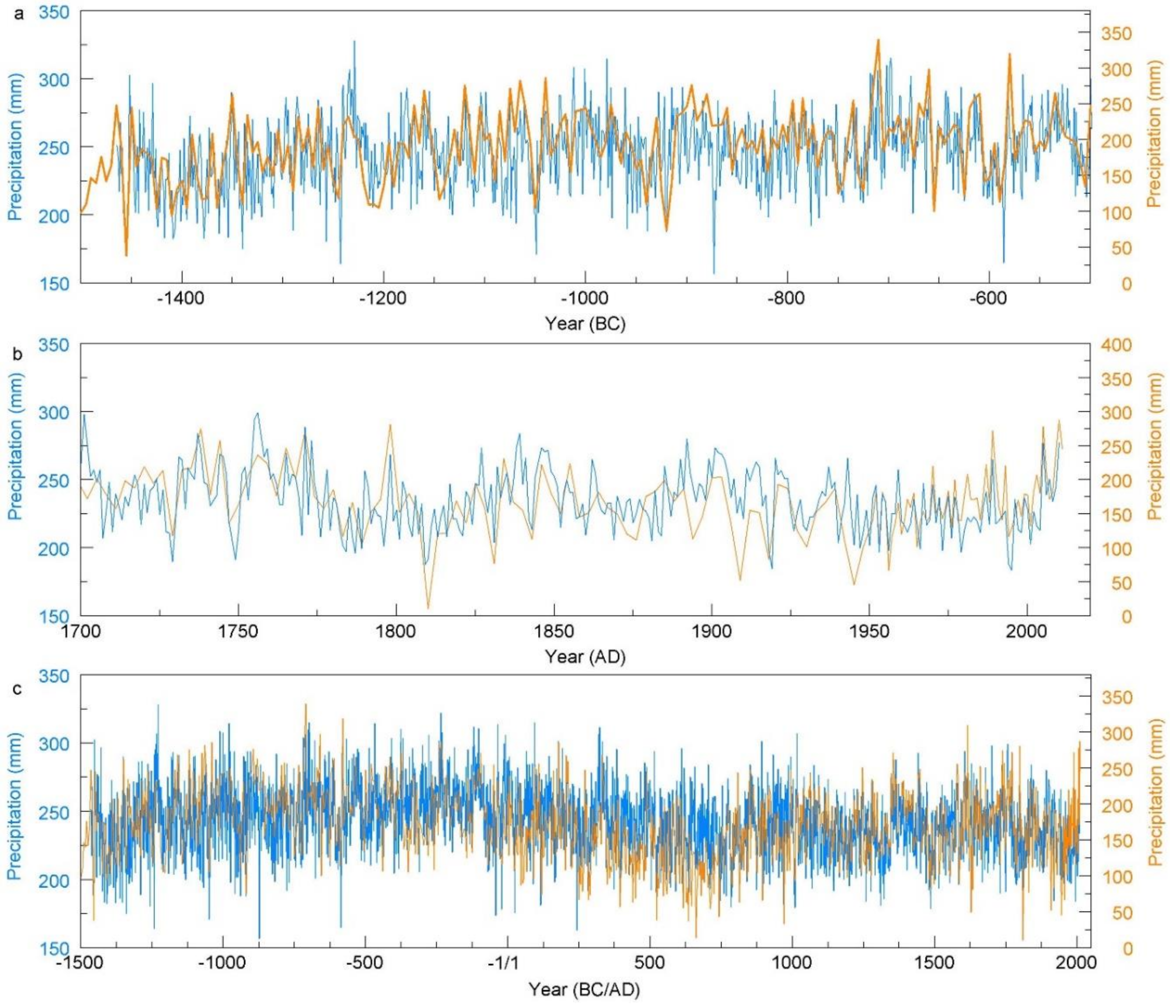

Supplementary Fig. 3. **Comparison of tree-ring δ^{18}O-based precipitation reconstructions of this study (blue) and with the precipitation reconstruction by Yang et al. (2021)[6] (orange) for the periods 1466 –500 BC (a), AD 1800–2010 (b), and 1466 BC–AD 2010 (c), respectively.**

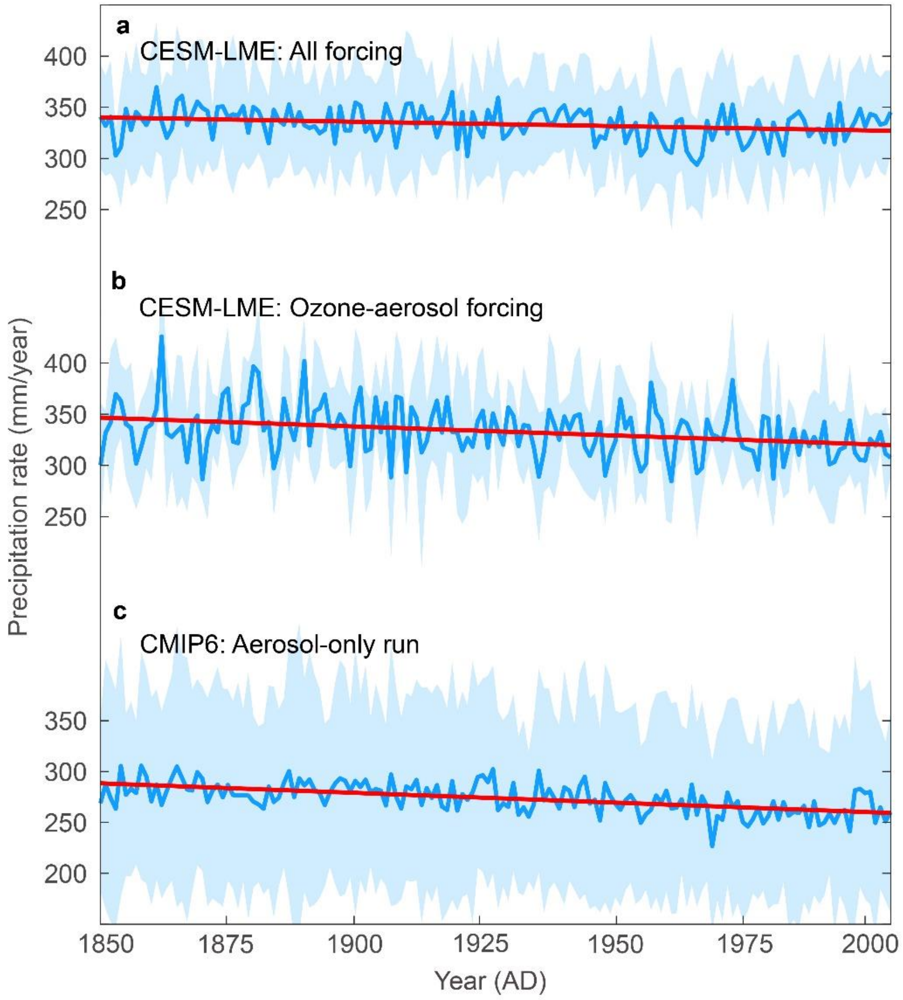

Supplementary Fig. 4. **Results of precipitation simulations of different models over the Tibetan Plateau (34–39°N, 94–102°E)**. **a,** Tibetan Plateau precipitation in CESM-LME full-forcing simulation; **b**, Tibetan Plateau precipitation in only Ozone-aerosol forcing simulation; **c**, Tibetan Plateau precipitation in CMIP6 multi-model aerosol-only forcing simulation. The blue line represents the mean, the shadow area represents mean ±1 standard deviation, and the red line represents the interannual trend.

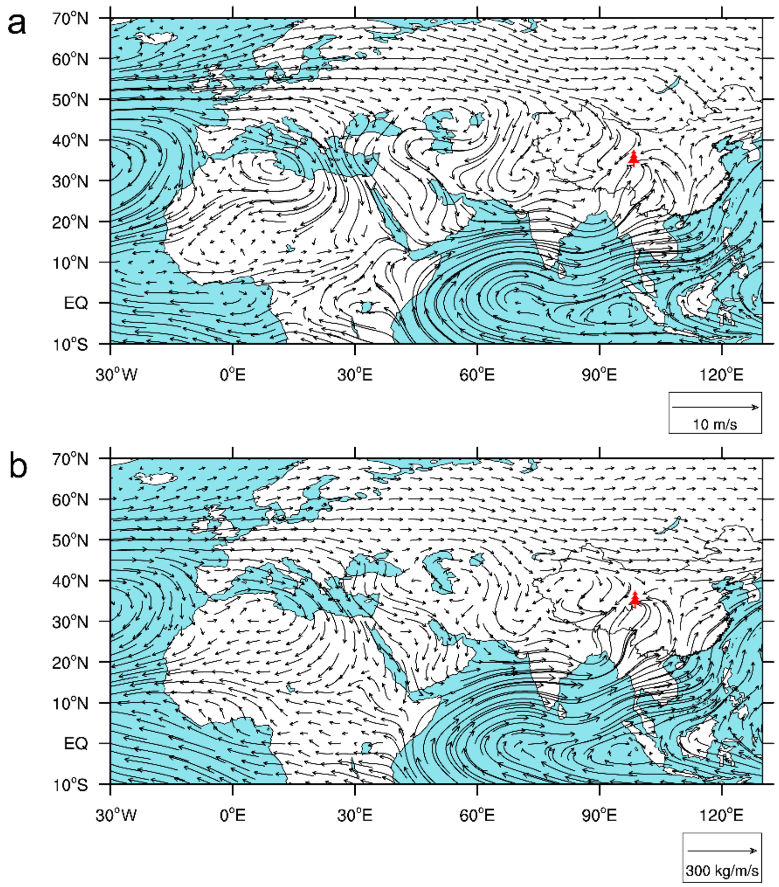

Supplementary Fig. 5. **Distribution of the mean wind field and the mean integral water vapor flux for the period June–July–August (summer) from AD 1979–2017. a**, Summer mean wind field (curly vectors, unit: m s^{-1}) at 850 hPa geopotential height. The data are from the National Center for Environmental Prediction & National Center for Atmospheric Research (NCEP/NCAR) Re-analysis 1 project[7]; **b**, Summer mean integrated water vapor flux field for the total air column (curly vectors, unit: kg m^{-1} s^{-1}) from ground to 300 hPa. The wind and specific humidity data originate from the NCEP/NCAR Re-analysis 1 project[7]. The maps reveal that most of the water vapor arriving at our study site during the summer months originate from South Asia and less from the Westerlies, indicating the overall importance of the South Asian summer monsoon for hydroclimate conditions in the study region. Additionally, there is a lower contribution of water vapor from the East Asian summer monsoon. Consequently, our reconstruction links to the Asian summer monsoon. The red tree symbol locates the Tibetan Plateau sample sites of this paper.

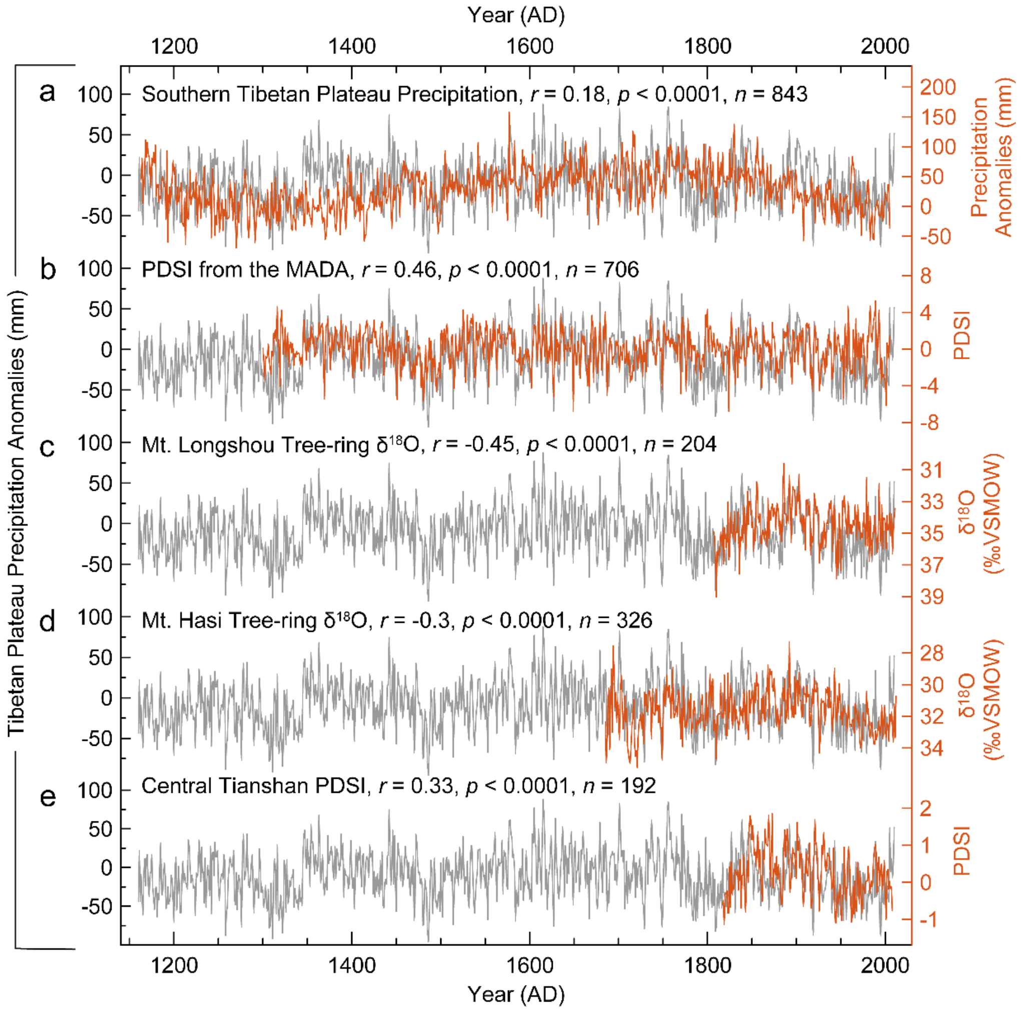

Supplementary Fig. 6. **Comparison and resulting correlation coefficients between our annual Tibetan Plateau precipitation reconstruction (grey lines in all panels) with various regional hydroclimatic indices from surrounding areas (orange). a**, Tree-ring $\delta^{18}O$-based precipitation reconstruction from the southern Tibetan Plateau[8] (representing the South Asia Monsoon, SAM); **b**, Gridded PDSI reconstruction (36.25°N, 98.75°E) from the Monsoon Asia Drought Atlas[9] (MADA) (representing the East Asian Summer Monsoon, EASM); **c**, Tree-ring $\delta^{18}O$ raw series from Mt. Longshou[10] (representing EASM variability; there is no precipitation reconstruction, but lower $\delta^{18}O$ values indicate wetter conditions, and *vice versa*.); **d**, Tree-ring $\delta^{18}O$ series from Mt. Hasi[11] (representing EASM variability; there is no precipitation reconstruction, but lower $\delta^{18}O$ values indicate wetter conditions, and *vice versa*.); **e**, Tree-ring $\delta^{18}O$-based PDSI reconstruction from Central Tianshan[12] (representing Westerlies).

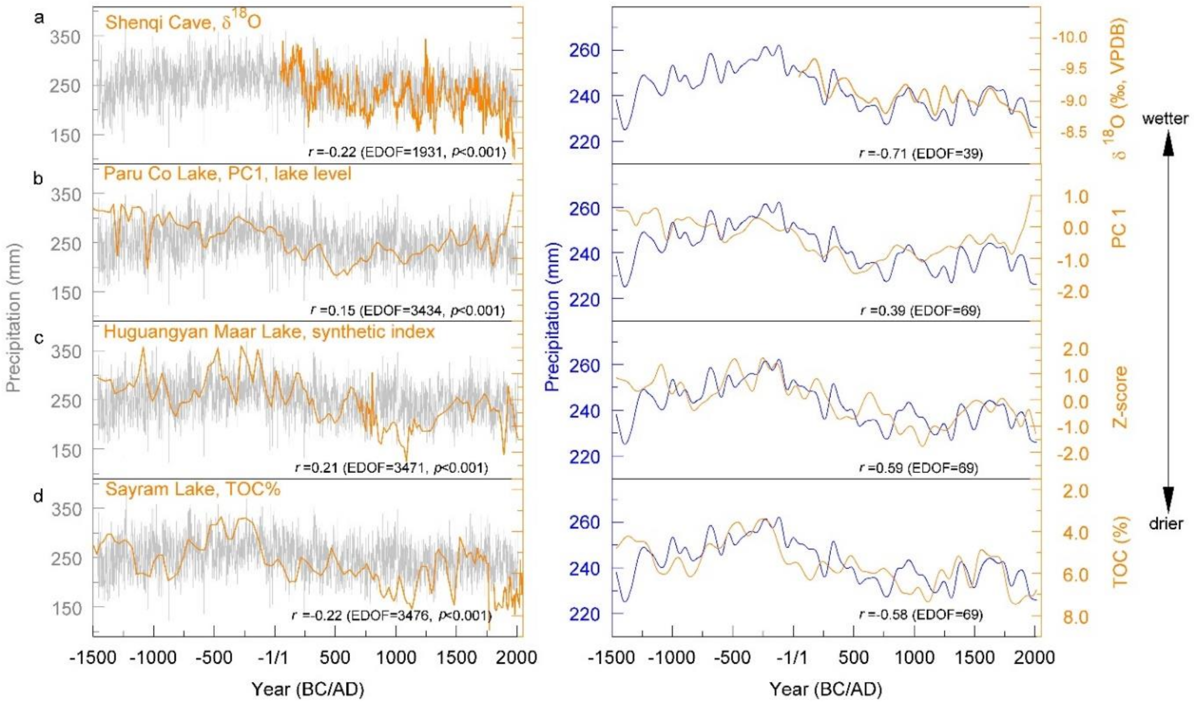

Supplementary Fig. 7. **Comparisons of our Tibetan Plateau precipitation reconstruction with different supra-regional hydroclimatic indices.** Left panels: Respective original data sets (grey lines represent Tibetan Plateau precipitation, and orange lines represent the other annually interpolated indices); Right panels: Filtered data assimilated with 100-yr filtering applied to the respective original data (blue lines represent Tibetan Plateau precipitation, and orange lines represent other indices). **a**, $\delta^{18}O$ records (AD 50–2010) of a stalagmite from Shenqi Cave, southeastern China[13] , with a lead time of 30 years (representing South Asian Monsoon (SAM), lower $\delta^{18}O$ values indicate wetter conditions, and *vice versa.*); **b**, PC1 of grain size variation of Paru Co Lake on the southeastern Tibetan Plateau, representing lake-level changes related to SAM activity[14]; **c**, Composite series from Sulfur ratios and percentage of tropical plants of Huguangyan Maar Lake, southeast China[15] (representing East Asian Summer Monsoon, EASM); **d**, Total organic carbon (%) in Sayram Lake sediments in west China[16], with a 90-yr time lag (Westerlies). EDOF gives effective degrees of freedom, and the significance confidence level was set at 95%.

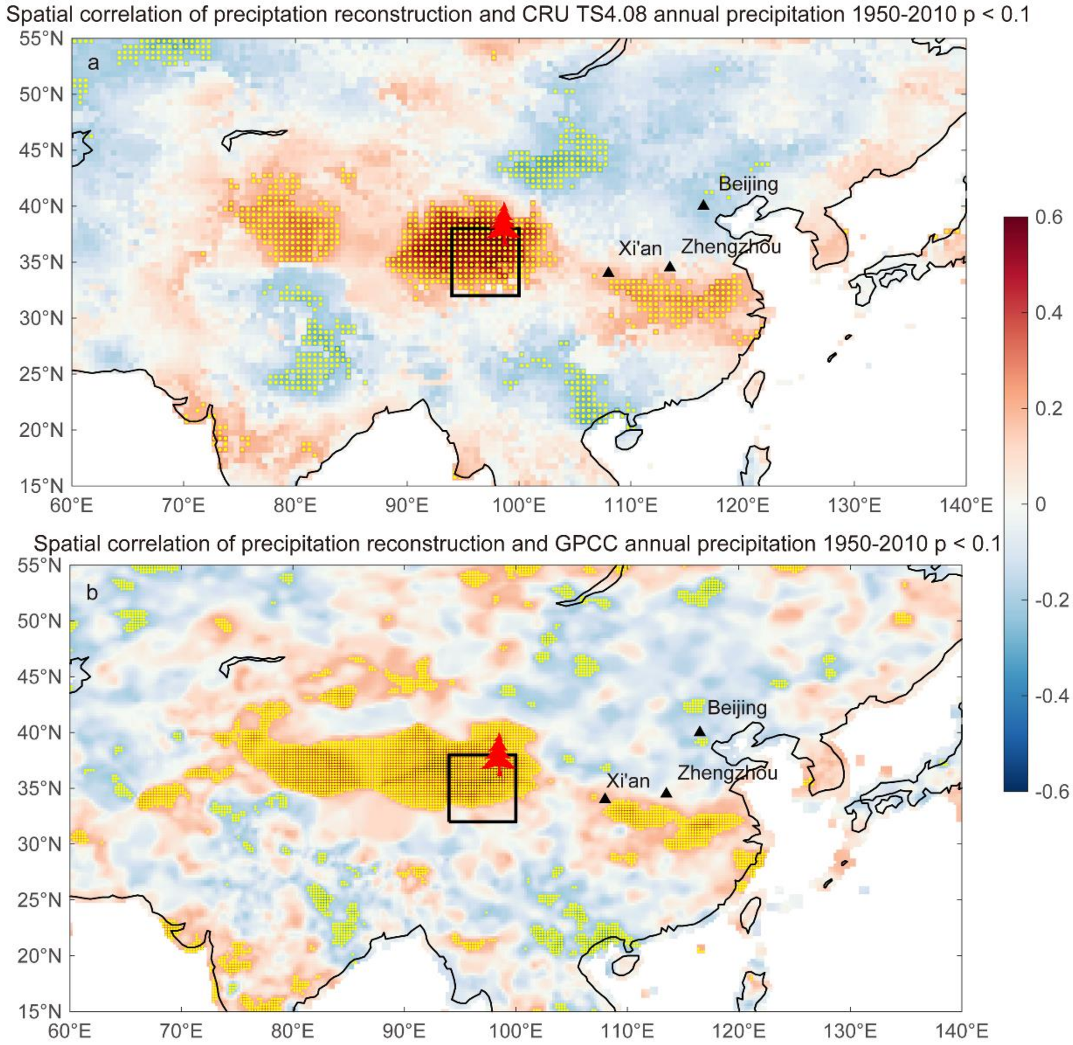

Supplementary Fig. 8. **Spatial correlation maps for the Tibetan Plateau precipitation reconstruction with interpolated precipitation datasets** a) Spatial correlation with data from the Climatic Research Unit (CRU) Time-Series (TS) version 4.08 of high-resolution gridded data (https://crudata.uea.ac.uk/cru/data/hrg/cru_ts_4.08/) and b) Spatial correlation with data from the Global Precipitation Climatology Centre (GPCC) (https://climatedataguide.ucar.edu/climate-data/gpcc-global-precipitation-climatology-centre).

The found correlation patterns confirm the high spatial representativity of our series with wide areas in High Asia including the TP and Central and eastern China. Correlations significant at the 95% confidence level are highlighted with yellow dotted grating.

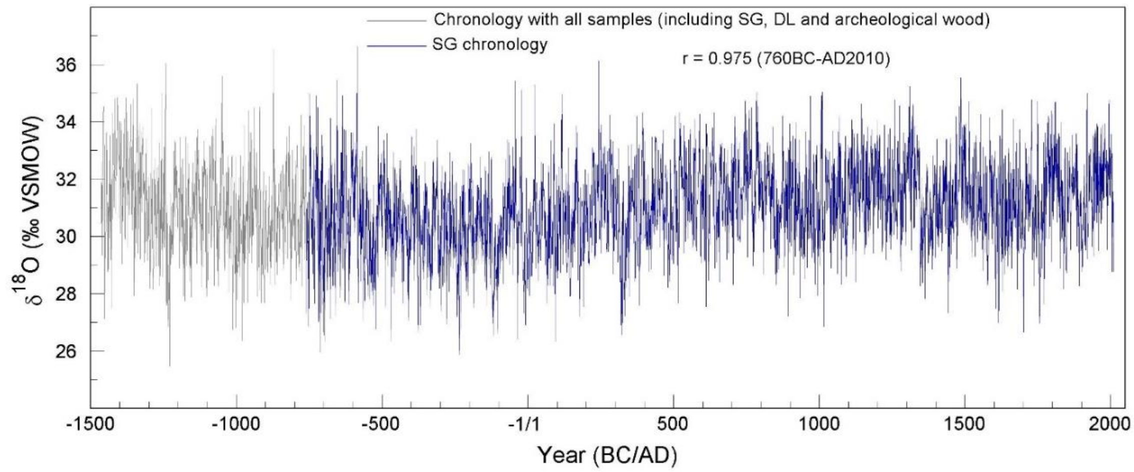

Supplementary Fig. 9. **Comparison between the tree-ring $\delta^{18}O$ chronology derived exclusively from the SG site including living and dead trees (blue line), and the overall $\delta^{18}O$ series encompassing all samples (gray line).**

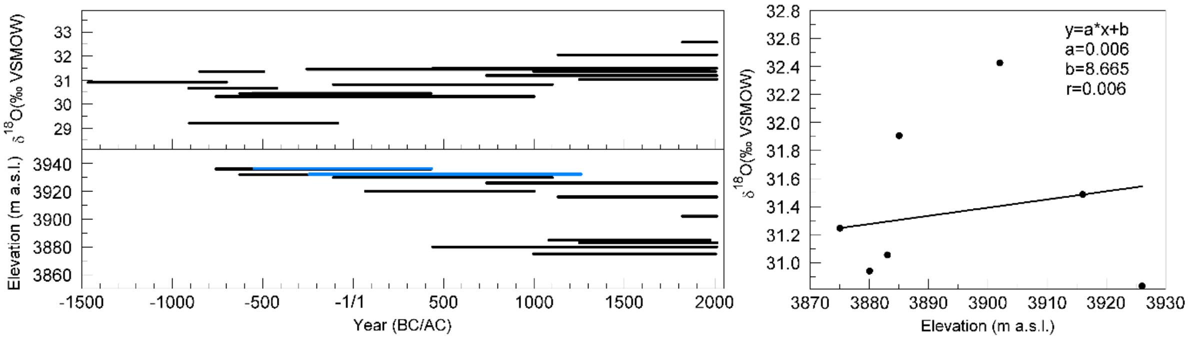

Supplementary Fig. 10. **Visualization of the relationship between average tree-ring δ^{18}O values from this study with altitude**. a, mean δ^{18}O value and lengths of each individual tree. b, elevations and lengths of each individual tree (blue lines indicating samples sharing the same elevation as other one), except for the archeological woods due to unknown true elevation. c, altitude-isotope relationship among the seven trees within the recent common period AD 1835–1974.

Supplementary tables

Supplementary Table 1. Information on tree-ring samples used for establishing the overall $\delta^{18}O$ chronology.

Site	Sample type	Samples (length)	Latitude, longitude	Altitude (m a.s.l.)
SG	Living trees	SG03a (AD 997–2004)<br>SG27a (AD 440–2010)<br>SG33c (AD 1251–2011)<br>SG49b (AD 1821–2010)<br>SG51b (AD 1134–2010)<br>SG79a (AD 737–2010)<br>SG73b (AD 69–1000)	37°28′N, 98°27′E	3800−4100
	Dead trees	SGd01 (110 BC–AD 1100)<br>SGd14 (257 BC–AD 1250)<br>SGd15 (628–81 BC)<br>SGd30 (761 BC –AD 102)<br>SGd31 (559 BC–AD 430)		
DL	Living tree	DL01 (AD 1082–1974)	36°03′N, 98°11′E	3900
XTT	Archaeological wood	XTT1_6 (849–496 BC)	37°16′N, 97°23′E	2900
DGM	Archaeological wood	DGM4_2 (907–87 BC)<br>DGM1_6 (1466–701 BC)<br>DGMd52 (911–424 BC)	37°25′N, 97°12′E	3300−3400

Supplementary Table 2. Significant correlation coefficients among all 17 individual δ^{18}O series used within this study (*p*<0.001). All illustrated correlations are very high and significant at the 99.9% confidence level, which means all the tree-ring δ^{18}O series show coherent variations. The blank cells in the table indicate no overlap between the two series. In each cell, the correlation coefficient is followed by the estimated effective number of degrees of freedom (EDOF)[17].

	SG49b	SG33c	SG51b	DL01	SG03a	SG79a	SG27a	SG73b	SG_d01	SG_d14	SG_d31	SG_d15	SG_d30	XTT1_6	DGM4_2	DGM_d52
SG33c	0.69/175															
SG51b	0.69/171	0.67/726														
DL01	0.54/139	0.49/708	0.48/810													
SG03a	0.56/169	0.68/718	0.58/822	0.39/846												
SG79a	0.70/175	0.76/710	0.64/806	0.56/841	0.53/938											
SG27a	0.32/165	0.67/710	0.55/814	0.48/854	0.50/946	0.68/1171										
SG73b						0.69/220	0.57/488									
SG_d01				0.75/19	0.51/100	0.65/346	0.52/624	0.60/843								
SG_d14			0.67/113	0.52/167	0.41/250	0.65/494	0.70/785	0.69/842	0.61/1176							
SG_d31								0.73/338	0.63/536	0.75/660						
SG_d15									0.69/29	0.70/167	0.56/465					
SG_d30								0.75/32	0.71/201	0.70/334	0.68/635	0.67/535				
XTT1_6											0.52/60	0.68/124	0.63/255			
DGM4_2									0.39/162		0.45/452	0.57/523	0.57/652	0.60/338		
DGM_d52											0.66/125		0.53/316	0.59/336	0.71/454	
DGM1_6													0.72/59	0.74/147	0.60/196	0.59/206

Supplementary Table 3. Correlations between the Tibetan Plateau tree-ring $\delta^{18}O$ chronology (this study) and observed monthly precipitation data starting from previous year (P) August until current year (C) October (for the period AD 1960–2010). The row label is the first month of the combination of months for the precipitation value, and the end month in the sequence of months in the correlation is given in the column label. Italic and bold numbers denote correlations at or above the 99% significance levels. "P" denotes months in the previous year, and "C" denotes months in current year of ring formation.

Start/end	P_{Jul}	P_{Aug}	P_{Sep}	P_{Oct}	P_{Nov}	P_{Dec}	C_{Jan}	C_{Feb}	C_{Mar}	C_{Apr}	C_{May}	C_{Jun}	C_{Jul}	C_{Aug}	C_{Sep}	C_{Oct}
P_{Jul}	-0.24	***-0.38***	***-0.47***	***-0.54***	***-0.53***	***-0.53***	***-0.53***	***-0.53***	***-0.54***	***-0.56***	***-0.69***	***-0.72***				
P_{Aug}		***-0.34***	***-0.48***	***-0.55***	***-0.53***	***-0.54***	***-0.54***	***-0.54***	***-0.56***	***-0.58***	***-0.69***	***-0.71***	***-0.77***			
P_{Sep}			***-0.41***	***-0.52***	***-0.50***	***-0.51***	***-0.50***	***-0.50***	***-0.53***	***-0.55***	***-0.65***	***-0.69***	***-0.77***	***-0.78***		
P_{Oct}				-0.30	-0.26	-0.27	-0.26	-0.27	-0.31	***-0.35***	***-0.57***	***-0.62***	***-0.73***	***-0.75***	***-0.72***	
P_{Nov}					0.14	0.05	0.06	0.01	-0.10	-0.14	***-0.51***	***-0.57***	***-0.69***	***-0.71***	***-0.68***	***-0.67***
P_{Dec}						-0.09	-0.02	-0.07	-0.16	-0.18	***-0.52***	***-0.57***	***-0.69***	***-0.71***	***-0.68***	***-0.67***
C_{Jan}							0.03	-0.03	-0.15	-0.17	***-0.53***	***-0.58***	***-0.69***	***-0.70***	***-0.68***	***-0.67***
C_{Feb}								-0.09	-0.18	-0.19	***-0.54***	***-0.58***	***-0.70***	***-0.71***	***-0.68***	***-0.67***
C_{Mar}									-0.17	-0.18	***-0.53***	***-0.57***	***-0.70***	***-0.71***	***-0.68***	***-0.67***
C_{Apr}										-0.10	***-0.52***	***-0.56***	***-0.69***	***-0.70***	***-0.67***	***-0.66***
C_{May}											***-0.50***	***-0.56***	***-0.69***	***-0.70***	***-0.67***	***-0.66***
C_{Jun}												***-0.41***	***-0.61***	***-0.65***	***-0.61***	***-0.60***
C_{Jul}													***-0.45***	***-0.47***	***-0.43***	***-0.43***
C_{Aug}														-0.26	-0.27	-0.26
C_{Sep}															-0.16	-0.15
C_{Oct}																-0.01

Supplementary Table 4. Statistics for the split calibration-verification test for the final precipitation reconstruction ranging from previous September to current August (hydrological year) on the Tibetan Plateau during AD 1961–2010.

	Calibration				Verification				
Period (AD)	*r*	*ST*	*t*	Period (AD)	*r*	*RE*	*CE*	*ST*	*t*
1961–1990	-0.75**	23+/7-**	3.62	1991–2010	-0.85**	0.62	0.56	16+/4-**	3.03
1981–2010	-0.80**	21+/9-*	3.74	1961–1980	-0.76**	0.55	0.35	9+/11-	3.49
1961–2010	-0.78**	41+/9-**	4.65						

r, correlation coefficient; *RE*, reduction of error; *CE*, coefficient of efficiency; *ST*, sign test.
* Significant at the 95% level.
** Significant at the 99% level.

Supplementary references

1. Nakatsuka, T. et al. A 2600-year summer climate reconstruction in central Japan by integrating tree-ring stable oxygen and hydrogen isotopes. *Clim. Past* **16**, 2153–2172 (2020).
2. Naulier, M. et al. A millennial summer temperature reconstruction for northeastern Canada using oxygen isotopes in subfossil trees. *Clim. Past* **11**, 1153–1164 (2015).
3. Büntgen, U. et al. Recent European drought extremes beyond Common Era background variability. *Nat. Geosci.* **14**, 190–196 (2021).
4. Shao, X. et al. Climatic implications of a 3585-year tree-ring width chronology from the northeastern Qinghai-Tibetan Plateau. *Quat. Sci. Rev.* **29**, 2111–2122 (2010).
5. Li, Q. et al. Delayed warming in Northeast China: Insights from an annual temperature reconstruction based on tree-ring $\delta^{18}O$. *Sci. Total Environ.* **749**, 141432 (2020).
6. Yang, B. et al. Long-term decrease in Asian monsoon rainfall and abrupt climate change events over the past 6,700 years. *Proc. Natl. Acad. Sci. U.S.A.* **118**, e2102007118 (2021).
7. Kalnay, E. et al. The NCEP/NCAR 40-year reanalysis project. *Bull. Am. Meteorol. Soc.* **77**, 437–472 (1996).
8. Grieβinger, J., Bräuning, A., Helle, G., Thomas, A. & Schleser, G. Late Holocene Asian summer monsoon variability reflected by $\delta^{18}O$ in tree-rings from Tibetan junipers. *Geophys. Res. Lett.* **38**, L03701 (2011).
9. Cook, E. R. et al. Asian monsoon failure and megadrought during the last millennium. *Science* **328**, 486–489 (2010).
10. Li, Q. et al. East Asian Summer Monsoon moisture sustains summer relative humidity in the southwestern Gobi Desert, China: Evidence from $\delta^{18}O$ of tree rings. *Clim. Dyn.* **52**, 6321–6337 (2019).
11. Wang, Y. et al. An Asian Summer Monsoon-related relative humidity record from tree-ring $\delta^{18}O$ in Gansu Province, north China. *Atmosphere* **11**, 0984 (2020).

12. Xu, G. et al. Drought history inferred from tree ring $\delta^{13}C$ and $\delta^{18}O$ in the central Tianshan Mountains of China and linkage with the North Atlantic Oscillation. *Theor. Appl. Climatol.* **116**, 385–401 (2014).

13. Tan, L. et al. High resolution monsoon precipitation changes on southeastern Tibetan Plateau over the past 2300 years. *Quat. Sci. Rev.* **195**, 122–132 (2018).

14. Bird, B. W. et al. A Tibetan lake sediment record of Holocene Indian summer monsoon variability. *Earth Planet. Sci. Lett.* **399**, 92–102 (2014).

15. Wang, X. et al. Millennial-scale Asian summer monsoon variations in South China since the last deglaciation. *Earth Planet. Sci. Lett.* **451**, 22–30 (2016).

16. Lan, J. et al. Late Holocene hydroclimatic variations and possible forcing mechanisms over the eastern Central Asia. *Sci. China Earth Sci.* **62**, 1288–1301 (2019).

17. Yan, H., Zhong, M., & Zhu, Y. The determination of degrees of freedom for digital filtered time series - an application in the correlation analysis between length of day variation and SOI. *Acta Astronomica Sinica.* **44**, 324–329 (2003).
